# Improving caregiver preparedness in the care transition of stroke patients: a scoping review

**DOI:** 10.25122/jml-2023-0142

**Published:** 2023-12

**Authors:** Mukhripah Damaiyanti, Haeril Amir, Desy Dwi Cahyani, Nurun Salaman Alhidayat, Novi Afrianti, Cut Rahmiati, Hera Hastuti, Apriani Susmita Sari, Rahmat Hidayat

**Affiliations:** 1 Faculty of Nursing, Universitas Muhammadiyah Kalimantan Timur, Kalimantan Timur, Indonesia; 2 Faculty of Public Health, Universitas Muslim Indonesia, Makassar, Indonesia; 3 Midwifery Department, Poltekkes Kemenkes Malang, Malang, Indonesia; 4 DIII Nursing, Institut Ilmu Kesehatan Pelamonia, Makassar, Indonesia; 5 Faculty of Nursing, Universitas Syiah Kuala, Aceh, Indonesia; 6 Akademi Keperawatan Kesdam Iskandar Muda Banda Aceh, Aceh, Indonesia; 7 Sekolah Tinggi Ilmu Kesehatan Fatmawati, Jakarta, Indonesia; 8 STIKes Hamzar Lombok Timur, Lombok, Indonesia

**Keywords:** caregiver, family caregivers, stroke, transition, care, readiness

## Abstract

Stroke is a life-threatening condition caused by the rupture of a brain blood vessel, potentially causing brain damage within minutes, severe disability, and even death. After initial hospitalization and rehabilitation, most stroke survivors return home, relying on their family members as caregivers for activities of daily living and treatments. This scoping review aimed to evaluate caregivers' readiness for transitioning stroke patient care to a home environment. We conducted a comprehensive search on Scopus, PubMed, ScienceDirect, and Google Scholar databases, followed by a secondary search to identify articles based on predefined criteria. A total of 14 articles were synthesized, leading to a series of findings: (1) there is a need to assess and identify caregiver needs, (2) the process needs relevant information, (3) caregivers are involved in the care process until the patients are discharged from the hospital, and (4) there is a need to offer support to caregivers. These results indicate that implementing strategies to enhance caregiver preparedness is crucial for the effective home-based care of stroke survivors.

## INTRODUCTION

According to the World Health Organization (WHO), stroke is ranked as the second leading cause of death worldwide after cardiovascular disease. Stroke happens when a blood vessel in the brain bursts, leading to brain damage within minutes, severe disability, and even death. In the United States, stroke is a prevalent cause of mortality, with the American Heart Association (AHA) reporting about 795,000 cases annually in individuals over 45 as of 2018 [1-3]. In Southeast Asia, the prevalence of stroke reached 11 million people each year. Indonesia, in particular, recorded a stroke prevalence of 10.9% in 2018 [4].

Following stroke, patients often experience prolonged impact [4]. Motor and sensory impairment, verbal, cognitive, and emotional changes, as well as bladder dysfunctions, are common after-effects of stroke and can significantly affect the quality of life, both in acute care settings and during at-home treatment [5, 6]. Therefore, stroke patients require ongoing comprehensive hospitalization, rehabilitation, and care from health providers and caregivers to deal with their daily living difficulties in mobility, self-care, and managing communication, but also their cognitive impairment, depression, and personality changes.

Caregivers are family members or hired professionals responsible for regularly looking after sick patients and meeting their needs [7]. According to the Emblem Health & National Alliance for Caregiving (2010), there are formal caregivers (professional workers) and informal caregivers (volunteers) [8, 9]. In Indonesia, caregiving is predominantly undertaken by family members, reflecting the cultural emphasis on extended family support [10]. However, this responsibility often leads to caregiver stress, anxiety, financial strain, and health and social welfare issues, especially when their needs are overlooked during the patient's recovery process [11-13]. More than 40% of caregiver families experience emotional stress and excessive burden [14]. Therefore, it is necessary to prepare the families to better navigate the recovery process, as well as the financial and social fallout of a stroke. Assessing the caregivers’ preparedness before the patient returns home is important to avoid psychological distress [15, 16]. The improvement in caregiver preparedness starts from the patient’s nursing care and rehabilitation and continues upon the return to a home environment (care transition back into the community).

Numerous studies have highlighted a significant gap in the readiness of family caregivers to effectively manage and maintain the optimal health of stroke patients. Research findings indicate that caregivers frequently report a lack of preparedness for post-discharge care responsibilities, primarily attributed to insufficient knowledge and skills [17]. This issue is further compounded by the fact that caregivers often need to seek out information and assistance independently without adequate guidance or support [18].

Previous research underscores the importance of evaluating caregiver preparedness and implementing appropriate strategies to enhance readiness [19]. Systematic reviews and meta-analyses revealed that health coaching strategies for caregivers are essential during the transition process to improve the health of stroke survivors [20]. Some authors evaluated the challenges and opportunities of caregivers after returning home [21]. Qualitative research pinpoints the importance of systematic communication during care in supporting the transition and improving knowledge and skills for caregivers [21]. The literature also delved into the preparedness and coaching strategies for caregivers, as well as challenges faced upon return to a home environment. Despite the existing research on the challenges faced by family caregivers of stroke patients, there is a notable gap in the literature regarding a comprehensive summary and conclusion on strategies to enhance caregivers' preparedness for transitioning from inpatient rehabilitation to home care. This gap justifies the need for research that consolidates and summarizes findings on this topic. This review aimed to identify and outline practical strategies that can improve the readiness of stroke caregivers for this critical transition.

## MATERIAL AND METHODS

### Data sources and search strategies

This scoping review followed the Preferred Reporting Items for Systematic Reviews and Meta Analyses (PRISMA) guidelines [22]. The review focused on identifying articles relevant to improving stroke caregivers' preparedness to transition from inpatient care to home-based rehabilitation. The time frame for the literature search was between 2010 and 2020. Articles were identified using several databases, namely Scopus, PubMed, ScienceDirect, and Google Scholar. A secondary search was conducted on the references list from the previously obtained articles. The Medical Subject Heading (MeSH) used in the search followed the PCC framework (population, concept, context) (Table 1).

**Table 1:** MeSH and search methods

Combination Word	Population ‘AND’	Concept ‘AND’	Context ‘AND’
OR OR OR OR OR	1. Stroke caregiver 2. Stroke Family 3. Stroke Caregiving 4. Stroke Careers 5. Combination 1-4 using ‘OR’	6. Transition 7. Transitional 8. Care Transition 9. Preparedness 10. Combination 6-9 using ‘OR’	11. Inpatient 12. Hospital care 13. Combination 11-12 using OR’
Next, combine the steps 5+10+13 together by using the conjunction ‘AND’			

### Identifying relevant sources

In this literature review, the initial screening process involved evaluating titles and abstracts of articles in Indonesian and English, focusing on the preparedness of stroke caregivers and strategies for improvement during the transition from inpatient rehabilitation to home care. The selection of articles was further refined based on the year of publication and the nature of the research (original or non-original research). Duplicates were excluded, and the results were presented. The inclusion criteria for the literature review were articles (a) focusing on the population of family caregivers of stroke patients, (b) addressing the concept of improving transition preparedness, (c) research set in the context of hospitalization or acute care, and (d) articles published internationally.

### Data extraction

After selecting articles that matched the research topic, data were selected for relevance, discussed, analyzed descriptively, and presented in the synthesis table.

**Figure 1:**
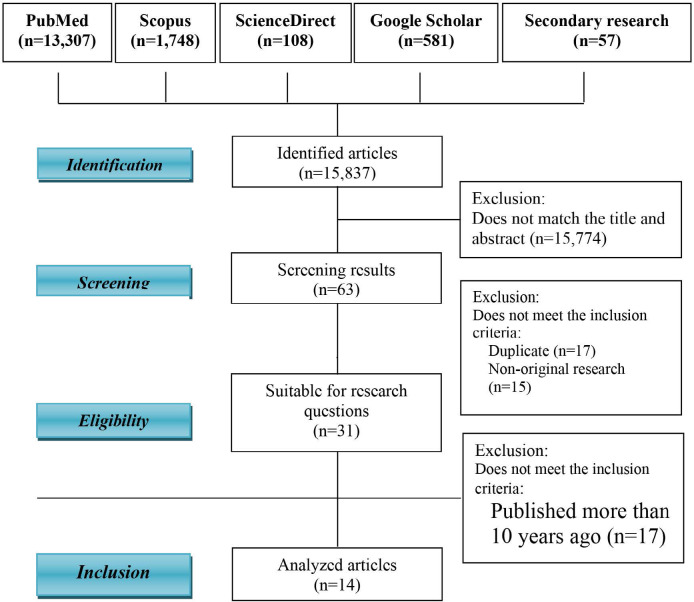
Search algorithm

After searching the four databases, 15,837 articles were identified and filtered based on the research population and the research objectives (from the abstract and article title). After the screening phase, 63 articles matched the research topic. The second screening was carried out to assess the suitability of the articles based on predetermined inclusion and exclusion criteria. During this phase, 17 articles were excluded because these were duplicates, and 15 due to non-original research. Ultimately, 14 articles matched the needs of this research and were further analyzed (Figure 1). The synthesis table includes authors, population and sample, objectives, research methods, interventions, and key findings [23-36] (Table 2).

**Table 2:** Grid synthesis for improving caregiver preparedness in the care transition of stroke patients

Author	Methodology	Aims/Purpose	Sample	Intervention	Study Outcomes	Key Finding
Cheng *et al.* [23]	Randomized controlled trial	To evaluate the effectiveness of a strength-oriented psychoeducational program on caregiving competence in improving readiness during home discharge.	One hundred and twenty-eight caregiver–survivor dyads with an age range of 19-80 years.	Respondents were randomly divided into the intervention and control groups for 26 weeks. The intervention consisted of two structured individual face-to-face pre-discharge education sessions.	Providing psychoeducational interventions to caregivers relates to a significant increase in family functioning and social support.	There was a significant effect on caregiver readiness after returning home.
Lutz *et al.* [24]	Qualitative research with a grounded theory approach	To develop theories in improving stroke caregiver preparedness and outcome and identify gaps in caregiver preparedness.	Forty family caregivers who cared for 33 stroke patients. The caregivers were female, consisting of 27 dyads, 12 adult children/in-laws, and one mother, with an age range of 23-89 years.	Interviews with inpatient caregivers up to 6 months after treatment. The interviews lasted 60-90 minutes and used audio recordings and manuscripts.	To (1) conduct patient and caregiver risk assessments, (2) identify and prioritize gaps between patient needs and caregiver commitment and capacity, and (3) develop interventions to improve caregiver preparedness.	Improving stroke caregivers’ preparedness for the transition from inpatient rehabilitation to home.
Hughes *et al.* [25]	Descriptive qualitative research	To improve the transition between caregivers and stroke patients.	Thirty-five stroke survivors and 20 family caregivers were recruited from three regional hospitals during their hospitalization discharge.	The participants were divided into three groups, with two meetings at two research locations during hospitalization. Upon home discharge, patients were randomized into one of three study arms –usual care (Group 1), SWCM (Group 2), or SWCM plus access to the study website (Group 3).	(1) Emotional, medical, financial, and role support, (2) health information, and (3) involvement in care.	Development of Social Work Case Management (SWCM) interventions to improve caregiver preparedness.
Hall *et al.* [26]	Systematic review and qualitative research	To develop a program plan for an evidence-based intervention to reduce the burden on the careers of stroke survivors and ensure caregivers caregivers’ preparedness.	Two systematic reviews and 33 family caregivers recruited in the inpatient stroke unit were asked qualitative interview questions.	The intervention consisted of six stages, where the first four stages of the Intervention Mapping were applied. The intervention development process (1) needs assessment, (2) identification of results and objectives, (3) selection of theoretical methods and practical applications, and (4) creation of program plans.	(1) Preparing capacity as a caregiver, (2) offering support for health, emotional, and practical services, (3) offering information on skills and roles as a caregiver, and (4) involvement in care.	Relevant interventions in improving caregiver preparedness before and after returning home.
Tseung *et al.* [27]	Descriptive qualitative research	To explore factors affecting the implementation of caregiver support programs in healthcare institutions.	Stakeholders from 11 regions with 43 professional nurses.	Data collection was obtained by interviews conducted for four months until saturation.	Support strategy implementation for caregivers to improve preparedness in caring for stroke patients.	Implementation strategy for caregivers.
Cameron *et al.* [28]	Descriptive qualitative research	To explore patient and caregiver during hospitalization and their role in the home discharge process	Fifteen female caregivers consisting of 10 parents, three dyads and two friends with an age range of 41 years. Thirty-one caregivers received standard care (n=10), self-directed (n=10), or stroke support person-directed (n=11) interventions.	The interviews were carried out consecutively for a week after the first WP and the second was carried out approximately four weeks after the patient returned home.	Improving caregiver preparedness by (1) supporting roles, activities, emotional, and financial needs, (2) offering information about care, rehabilitation, and skills.	Improve caregiver preparedness.
Giosa *et al.* [29]	Qualitative research with a grounded-theory approach	To develop caregiver understanding and experience in supporting the care transition between hospital and home and offering support during the care transition.	Twelve family caregivers were recruited, of which only two were caregivers for a stroke patient (the patient’s wife and son).	The semi-structured interview was conducted for 44 minutes. Researchers used a combination of scripted and spontaneous questions to capture the complexity of the participants’ transitional care experiences, perceptions of needs, challenges, and self-reported preparedness for their new role.	Components characteristic of caregiver needs during care transitions include (1) assessment of the unique family situation, (2) practical information, education, and training, (3) involvement in the planning process, (4) agreement between formal and informal caregivers, (5) time to make arrangements in personal-life, (6) emotional readiness.	Needs and readiness of caregivers in caring for stroke patients after returning home.
Cameron *et al.* [30]	Mixed methods research with Single-blind, randomized controlled trial sample selection.	To obtain guidelines for family support programs (TIRSFSP) and education to enhance caregiver readiness and emotional well-being.	Three hundred participants were recruited. They were randomly divided into three groups, with 100 participants in each group.	The participants were divided into three groups: (1) TIRSFSP, guided by professional nurses in the field of stroke during treatment up to 12 months after stroke (2) independent TIRSFSP caregivers who understand stroke (3) standard care. Further, the participants were assessed at 3,6 and 12 months post-stroke.	Timely education and support for caregivers from nurses is needed in the rehabilitation process to increase caregiver readiness.	Development of interventions to improve caregiver readiness.
Creasy *et al.* [31]	Qualitative research with a grounded theory approach	To explore how caregivers are involved in rehabilitation settings.	Interviews were conducted with 17 family caregivers.	The semi-structured interview was conducted and audio recorded, transcribed verbatim, and verified for accuracy.	Caregivers need information as they prepare for the stroke survivors’ eventual discharge home, focusing on prognosis for recovery, physical care provision, medication administration, diet, and post-discharge therapy, as well as involvement in care and decision-making.	Healthcare responsibilities involve caregivers in inpatient rehabilitation, which can increase their knowledge and preparation after returning home.
Day *et al.* [32]	Randomized clinical trial	To describe an educational intervention focused on family caregivers of stroke survivors for the development of home care.	Forty-eight family caregivers (wives) >18 years.	Participants were divided into two groups: intervention group (n=24) and control group (n=24). The participants in the intervention group were visited over one month.	Family caregivers need to ensure comfort, improved knowledge, and better skills in emotional management.	Support and knowledge needs.
Young *et al.* [33]	Qualitative research with a grounded theory approach	To understand the needs of spousal caregivers of stroke survivors during the transition from rehabilitation to home.	Fourteen spousal caregivers (wives) with an average age of 63 (49-82).	Semi-structured interviews were conducted pre- and post-discharge from rehabilitation as part of a larger study that focused on identifying caregiver and stroke survivor needs as they transitioned home from inpatient rehabilitation.	A comprehensive, systematic caregiver assessment should be conducted. The assessment reviewed commitment, capacity, and preparedness to provide care.	Family members are prepared to take on the caregiving role.
Perrin *et al.* [34]	Randomized Control Trial	To determine the level of satisfaction of caregivers and stroke patients after being given educational interventions and problem-solving support.	Sixty-one female family caregivers consisting of 43 wives, three mothers, 10 daughters, and four relatives with an average age of 58 years.	Participants were divided into two groups: the control and intervention groups. Further evaluations were carried out after three months.	After being given an intervention providing education, skills, and problem-solving information, the level of depression and tension in caregivers decreases.	There was a decrease in depression and tension after the intervention.
Gustafsson & Bootle [35]	Qualitative research	To enhance the understanding of the transition experience for patients with stroke and their careers during discharge and the first month at home.	Five family caregivers, including two male dyads, one female dyad, one female child, and one friend, with an age range of 36-79 years.	Five participants completed semi-structured interviews one month following discharge by exploring preparation and transition experiences.	Caregivers need information and rehabilitation services, and support in carrying out their roles and responsibilities as caregivers.	Caregivers need information and rehabilitation services.
Ghazzawi *et al.* [36]	Descriptive Qualitative research	To examine family caregivers’ perceptions and experiences navigating the stroke rehabilitation system.	Fourteen participants with an average age of 63 years.	Interviews of 30-45 minutes were conducted in person or by telephone using a semi-structured interview guide.	The health system should provide information and access to support in the community to meet the needs of stroke patients and caregivers.	Caregivers need information and support during the transition home.

## RESULTS

Fourteen articles consistent with the research topic were selected. The studies were conducted in several countries, such as the United States, the United Kingdom, Canada, Brazil, Australia, and China. The methodologies used in these studies varied, encompassing quantitative, qualitative, and mixed methods approaches. Specifically, ten of these articles used qualitative methods with a grounded theory approach, three employed quantitative research techniques, and one was based on a mixed-methods study.

The articles selected for this study included various caregiver respondents (Table 3). One article focused on professional caregivers and their experiences transitioning from inpatient care to home settings. However, most caregivers in the studies were family members (e.g., wife, husband, and children).

**Table 3:** Characteristics of caregiver respondents

Caregiver	Total	Age	Transition
Wife	99	20-40	To home
Husband	7		To home
Mother	14		To home
Daughter	23		To home
Son	1		To home
Female friends	7		To home
Family caregiver	415		To home
Professional Caregiver	43		Rehabilitation

The literature review identified four key findings, emphasizing crucial aspects of caregiver support in stroke patient care. These include (a) the need to assess and identify caregiver preparedness, (b) the necessity of providing caregivers with relevant information, (c) the importance of caregiver involvement up until the patient's preparation for discharge, and (d) the overall support required by caregivers [23-27, 29-35, 37] (Table 4). Furthermore, Creasy *et al*. [31] also explained that caregivers need financial support from family members or other relatives. In addition, caregivers also need significant support in managing emotions [30].

**Table 4:** Main findings identified

Authors and articles	Key findings	Main theme
Oliva-Moreno *et al.* [37]; Lutz *et al.* [24];Young *et al.* [33]; Cameron *et al.* [30]; Giosa *et al.* [29]	• Caregivers’ commitment • Caregivers’ capacity • Environment preparedness • Emotional readiness (depression and psychosocial considerations) • Conceptual knowledge of disease	Assess and identify caregiver preparedness
Cameron *et al.* [30]; Day *et al.* [32]; Giosa *et al.* [29]; Hall *et al.* [26], Gustaffson *et al.* [35]; Creasy *et al.* [31] and Perrin *et al.* [34]	• Skills (changing clothes, moving, toileting, and feeding) • Conceptual knowledge of disease • Role change • Treatment adherence • Stress management • Provision of relevant information and support	Provide relevant information and support
Giosal *et al.* [29]; Hall *et al.* [27];Creasy *et al.* [31]	• Changing clothes • Changing diapers • Toileting • Feeding • Organizing home discharge plans	Care participation until preparation for discharge
Cheng *et al.* [23], Tseung *et al.* [27], Cameron *et al.* [30], Hall *et al.* [26], Creasy *et al.* [31], Hughes *et al.* [25];	• Psychosocial support • Caring support • Knowledge • Financial sources	Supports for caregivers

## DISCUSSION

This review was conducted to summarize and describe concepts related to factors that need attention in the transition of care for stroke patients. The research identified several key aspects in this regard.

### Assessment and identification of caregiver preparedness

The caregivers’ preparedness must be assessed and identified early before patients return home. In this case, several factors influence caregiver readiness: commitment, capacity, and knowledge in providing care to patients’ families. In addition, to motivate and spiritually support the family members, a caregiver must have a commitment and capacity to carry out their role well [38]. The commitment includes maintaining the relationship with the patient, motivation in caregiving, and spiritual support, while their capacity as a caregiver refers to considering their health status, ability to maintain a role, financial resources, and knowledge [24]. In addition, environmental readiness is also one of the most common requirements for caregivers. Access is one component that aids caregivers in helping stroke patients recover [39]. This assessment was carried out to identify the caregivers’ needs and care plans that can improve the quality of the process and health as well as reduce expenses [17]. A previous study [23] highlighted the importance of initial assessments before patients return home. Such an assessment helps families to be better prepared for the caregiving role and to adapt to post-rehabilitation activities more effectively. These findings align with other research emphasizing the need to identify specific skills, commitment, capacity, and knowledge essential for caregivers in managing the care of family members [40, 41]. This perspective is further reinforced by Langa *et al*., who argue that caregivers should have the commitment, capacity, skills, and knowledge to improve the quality of life before the patient's return home [42]. The results of this assessment can direct health providers to deliver clear and relevant information according to patient needs.

### Relevant information needs

Caregivers need to be informed about stroke from inpatient care until preparation for discharge. This information should be adapted to their needs [43]. According to Yedidia *et al*. [44], patients and caregivers need information on available services, stress management, illness, and treatment. This is in line with research conducted by Alvarez *et al*. [45], who stated that a caregiver needs personalized information on treatment, nutrition, stress management, and ambulation to improve the quality of life for sick patients. Furthermore, Tsai *et al*. [46]. supported this statement by stating that providing relevant information and counseling caregivers can help them meet the needs of patients’ families. This information is provided from when the patient enters the hospital until the preparation to go home and is adjusted to the caregiver's needs [47]. Moreover, nurses have a vital role in providing information and knowledge during the transition period [48].

### Caregivers’ involvement during in-patient care

Long-term care for stroke patients with limited resources requires active caregiver involvement until patients return home. Caregivers need to know their duties and responsibilities (e.g., helping with meals, participating in rehabilitation activities, and actively participating in decision-making during the acute care phase) [49]. The involvement of caregivers in the hospital care process can help them learn to manage problems, increase knowledge, and reduce physical and emotional stress. This, in turn, has a positive impact on the health of stroke patients [50, 51]. This concept is supported by previous research, indicating that caregiver involvement in care and decision-making processes can enhance their overall experience, aiding them in being more prepared and improving the health of post-rehabilitation stroke patients [52]. Consequently, this active participation of caregivers can lead to more tailored and effective clinical interventions and rehabilitation plans.

### Support for caregivers

Increasing caregivers’ desire to care for stroke patients is also required to support the care during rehabilitation. The support given to caregivers from various parties can reduce the burden and increase their enthusiasm [53]. A study by Hekmatpou *et al*. underscores the importance of supporting caregivers in adapting to the changes that come with caring for a stroke patient. The research highlights that caregivers require emotional support, assistance from the patient's family, educational resources, and financial assistance to enhance the quality of life for both the caregiver and the patient [54]. This is in accordance with research conducted by Cameron *et al*., which showed that a caregiver needs knowledge, training, and psychosocial support to care for patients. This statement is further reinforced by Cemy *et al*. [55]. who suggest that caregivers need psychological, social, and spiritual support to care for their patients.

## CONCLUSION

This scoping review uncovered critical aspects of improving caregiver preparedness in the care transition for stroke patients. The findings reveal that close relatives are the most suited for enacting the caregiver role for stroke patients. However, a plan is needed to prepare families to care for patients during post-stroke rehabilitation. Several strategies can be carried out by a health provider to improve caregiver preparedness before patients return home, including (a) identifying and assessing caregivers' readiness to take on their roles, commitment, and capacity in their new position, (b) providing relevant information according to caregivers’ needs such as disease prevention processes, actions, post-stroke complications, treatment, stress management, and rehabilitation, (c) involving caregivers in the care and discharge planning of stroke patients to provide experience and knowledge of the activities to be carried out during the rehabilitation process, and (d) providing support to caregivers from health providers, friends, and relatives to decrease the negative effect and increase enthusiasm in providing care.

Therefore, healthcare workers should prioritize the preparation of caregivers before stroke patients are discharged home. This comprehensive approach involves assessing and identifying their needs, providing relevant information, getting involved in care and discharge planning, and supporting stroke patients at home.
